# Cyclophosphamide induces the loss of taste bud innervation in mice

**DOI:** 10.1093/chemse/bjae010

**Published:** 2024-02-29

**Authors:** Ryan M Wood, Erin L Vasquez, Krystal A Goyins, Eduardo Gutierrez Kuri, Kevin Connelly, Saima Humayun, Lindsey J Macpherson

**Affiliations:** Department of Neuroscience, Developmental and Regenerative Biology, The University of Texas at San Antonio, One UTSA Circle, San Antonio, TX, USA; The Graduate Program in Neuroscience, The University of Texas at San Antonio, San Antonio, TX, USA; Department of Neuroscience, Developmental and Regenerative Biology, The University of Texas at San Antonio, One UTSA Circle, San Antonio, TX, USA; Department of Neuroscience, Developmental and Regenerative Biology, The University of Texas at San Antonio, One UTSA Circle, San Antonio, TX, USA; The Graduate Program in Developmental and Regenerative Sciences, The University of Texas at San Antonio, San Antonio, TX, USA; Department of Neuroscience, Developmental and Regenerative Biology, The University of Texas at San Antonio, One UTSA Circle, San Antonio, TX, USA; Department of Neuroscience, Developmental and Regenerative Biology, The University of Texas at San Antonio, One UTSA Circle, San Antonio, TX, USA; Department of Neuroscience, Developmental and Regenerative Biology, The University of Texas at San Antonio, One UTSA Circle, San Antonio, TX, USA; Department of Neuroscience, Developmental and Regenerative Biology, The University of Texas at San Antonio, One UTSA Circle, San Antonio, TX, USA; Brain Health Consortium, The University of Texas at San Antonio, San Antonio, TX, USA

**Keywords:** taste, chemotherapy, cyclophosphamide, gustatory fiber

## Abstract

Many common chemotherapeutics produce disruptions in the sense of taste which can lead to loss of appetite, nutritional imbalance, and reduced quality of life, especially if taste loss persists after treatment ends. Cyclophosphamide (CYP), an alkylating chemotherapeutic agent, affects taste sensitivity through its cytotoxic effects on mature taste receptor cells (TRCs) and on taste progenitor cell populations, retarding the capacity to replace TRCs. Mechanistic studies have focused primarily on taste cells, however, taste signaling requires communication between TRCs and the gustatory nerve fibers that innervate them. Here, we evaluate cyclophosphamide’s effects on the peripheral gustatory nerve fibers that innervate the taste buds. Following histological analysis of tongue tissues, we find that CYP reduces innervation within the fungiform and circumvallates taste buds within 4 days after administration. To better understand the dynamics of the denervation process, we used 2-photon intravital imaging to visualize the peripheral gustatory nerve fibers within individual fungiform taste buds up to 20 days after CYP treatment. We find that gustatory fibers retract from the taste bud properly but are maintained within the central papilla core. These data indicate that in addition to TRCs, gustatory nerve fibers are also affected by CYP treatment. Because the connectivity between TRCs and gustatory neurons must be re-established for proper function, gustatory fibers should continue to be included in future studies to understand the mechanisms leading to chemotherapy-induced persistent taste loss.

## Introduction

The incidence of cancer and the rate of chemotherapy treatment is growing rapidly (Wilson et al. 2019). With this, the rise of chemotherapy-related side effects grows as well. Cancer patients (50% to 80%) who receive chemotherapy treatments experience taste alteration in the form of reduced taste sensitivity, distorted taste perception, or even complete loss of taste during treatment (Bernhardson et al. 2008; Zabernigg et al. 2010; Gamper et al. 2012a, 2012b). These deficits can persist for months beyond the end of therapy (Zabernigg et al. 2010). Indeed, the side effects of chemotherapy on taste are listed as one of the most disturbing elements of treatment, negatively impacting the patient’s ability to eat and drink and maintain healthy nutritional balance (Hutton et al. 2007). This can result in loss of appetite, malnutrition, poor recovery, and a reduced quality of life. Unfortunately, there are few, if any, effective treatments for those experiencing taste loss, partially due to major gaps in our understanding of the mechanisms leading to persistent taste dysfunction after cancer treatment.

Since many chemotherapy drugs target the fast dividing cancer cells, other proliferative cell types are also affected, most visibly causing hair loss, but also affecting the immune system, digestive system, and the highly proliferative taste progenitor cells within taste buds (Murtaza et al. 2017; Gaillard and Barlow 2021). Alkylating chemotherapy drugs such as cyclophosphamide (CYP) produce a 2-phase disturbance in taste behavior in mice—one within 24 h of drug administration, and a second which emerges 4 to 8 days later (Zabernigg et al. 2010; Mukherjee and Delay 2011; Mukherjee et al. 2013, 2017; Murtaza et al. 2017). The first phase is due to direct cytotoxic effects on the lingual epithelium causing an early loss of taste receptor cells (TRCs). Then, the second disturbance is due to the disruption of the taste stem cell population responsible for TRC renewal, temporarily retarding the system’s capacity to replace the TRCs (Zabernigg et al. 2010; Mukherjee and Delay 2011; Mukherjee et al. 2013, 2017). Once chemotherapy treatment ceases, the remaining population of unaffected taste stem cells can begin to repopulate the taste buds with new TRCs. The normal rate of turnover for TRCs in the taste bud is 10 to 20 days (Miura and Barlow 2010). In mouse models of CYP taste loss, most TRC populations recover 16 to 20 days after treatment (Mukherjee and Delay 2011; Mukherjee et al. 2013, 2017).

Another type of chemotherapy drug used most often to treat basal cell carcinomas, inhibits the hedgehog signaling pathway. Notably, these drugs, including vismodegib and sonidegib, produce a significant disturbance or complete loss of taste in most patients within 2 months after the initiation of treatment (Von Hoff et al. 2009; Sekulic et al. 2012; Tang et al. 2012). In this case, these drugs do not produce the initial die-off of TRCs but cause a reduction in new taste bud cells and delay TRC maturation (Castillo-Azofeifa et al. 2017, 2018; Lu et al. 2018). Regeneration of the TRCs after treatment depends on Sonic Hedgehog (Shh) signaling supplied by the gustatory neuron fibers (Castillo-Azofeifa et al. 2017, 2018; Lu et al. 2018). A careful analysis of chorda tympani fibers within fungiform taste buds after hedgehog pathway inhibition with sonidegib showed a significant effect on the innervation pattern within the papillae after the loss of TRCs (Donnelly et al. 2022). Compared to a normal fungiform papilla where gustatory fibers project into the bud interdigitating with the elongated taste receptor cells, after sonidegib-induced loss of the TRCs, the fibers project outward into a flat disc-shaped bundle that doubled in size (Donnelly et al. 2022). The authors suggest that maintenance of fibers near the sites of lost taste buds is important for regeneration, such that the continued presence of these fibers in the vicinity of regenerating taste buds promotes reinnervation of newly formed buds.

In any case, as TRCs repopulate within the taste buds, they must resume communication with peripheral gustatory sensory neurons innervating the tongue via synaptic contacts. If this rewiring process is successful, taste function will gradually return to baseline. However, the fact that some chemotherapy patients experience protracted taste disturbances (over months and years) indicates that this recovery and rewiring process is prone to failure (Hutton et al. 2007). To date, most research on mechanisms leading to chemotherapy-induced taste dysfunction has focused on the loss and repopulation of TRCs within taste buds. A critical element that is missing in these studies is understanding the contribution of gustatory sensory neurons in the initial loss and eventual re-establishment of taste function after chemotherapy treatment. Therefore, to monitor the morphology of gustatory nerve fibers innervating the taste buds and to determine if the innervation patterns change over the course of CYP treatment, we used a combination of immunohistochemistry and longitudinal intravital 2-photon imaging.

## Materials and methods

### Animals

All procedures were conducted in accordance with US National Institutes of Health (NIH) guidelines for the care and use of laboratory animals and were approved by the University of Texas at San Antonio IACUC. Both male and female mice were used in this study. For immunohistochemical experiments of sectioned tongue tissues, C57/b6 mice (Jax Strain #000664) were used along with Phox2b-Cre;tdTomato double transgenic mice (Jax Strains #016223, #007909). All mice were between 3 and 8 months old; weighing between 18 and 35g. Food and water were available ad libitum additionally after injection they were given access to a nutrient supplement (DietGel®) to aid recovery. The mouse colony was maintained on a regular 12/12 h light-dark cycle.

### Drug treatment

Cyclophosphamide solution was prepared immediately prior to a single IP injection by dissolving cyclophosphamide monohydrate powder (Cat. No. AC203960250, Fisher Scientific, Hampton, NH, USA) (Mukherjee and Delay 2011; Mukherjee et al. 2013, 2017) in sterile saline (0.9%) for a dose of 100 mg/kg. Control mice were injected with sterile saline based on their weight to match cyclophosphamide-injected mice. Mice were observed each day after injections through weighting and physical well-being checks.

### Sample collection

Mice were euthanized through CO_2_ inhalation followed by trans-cardial perfusion with 0.1 M PBS and 4% paraformaldehyde solution in 0.1 M PBS, sequentially. Tongues were extracted and soaked in 4% paraformaldehyde solution for 2 h at room temperature then cryoprotected in 30% sucrose at 4 °C overnight. The tongues were then dissected to separate the circumvallate papillae (CV) and the anterior 2/3 of the tongue housing the fungiform papillae. The anterior 2/3 of the tongue was then cut sagittal to split it into left and right halves. The left and right tongue tip and the CV were embedded in OCT compound and stored at –80 °C. Samples were cryo-sectioned at 15 μm thickness and mounted directly on slides.

### Immunostaining

Sections of the CV and left tongue tips were blocked in 10% donkey serum at 4 ^o^C for 1 h before they were incubated with primary antibodies: Krt8 (1:1000; DSHB Cat# TROMA-I, RRID:AB_531826) in 10% donkey serum in 0.1 M PBS overnight at 4 °C. After 3 × 5-min washes with PBS, secondary antibodies (Alexa Fluor® chicken488 RRID:AB_2340375) were added (1:1000) in 10% donkey serum in PBS and incubated overnight at 4 ^o^C. The slides were then washed for 3 × 5 min with PBS and then mounted for imaging. A Zeiss 710 confocal microscope was used to image the tongue sections. Z-stack images consisted of 15 stacks at 1 μm depth.

### Immunohistochemistry cell counting and quantification

For image analysis, ImageJ (FIJI) was used. Taste bud area profiles ROIs were determined by KRT8 staining and outlined (Supplementary Figure 1). KRT8 positive cells in each taste bud section were counted. Twenty buds per mouse were averaged for circumvallate tissues, and 10 taste buds were averaged for fungiform taste buds. Fungiform taste buds were selected within the first 5 mm of the anterior tip of the first 8 sections of the tongue. To analyze the gustatory nerve fibers, each image stack was converted to a maximum projection image. The fluorescence was adjusted to a median brightness value and a threshold was set to median brightness. An ROI of each bud was measured and converted from pixels to um giving us a fractional area of the gustatory fibers, allowing quantification of the total gustatory nerve fibers within the taste bud area profiles ROIs. For Phox2b;tdTomato immunohistology experiments, cyclophosphamide-injected mice were compared to saline-injected controls using the Mann–Whitney *U* test. Researchers were blinded to the condition of the mice during quantification and cell counting.

### Whole mount immunohistochemistry and quantification

The right tongue tips were thinned to the dorsal epithelial surface through the removal of the ventral muscle. The epithelium was placed in a 24-well plate and incubated with primary antibodies for Krt8 (1:500; DSHB Cat# TROMA-I, RRID:AB_531826) in 10% donkey serum in 0.1 M PBS for 4 days at 4 °C. After washing tissues 3× with PBS for 20 min, secondary antibodies (Alexa Fluor®rat488; Thermo Fisher Scientific, catalog # A-11006, RRID AB_2534074) were added (1:1000) in 10% donkey serum and incubated for 3 days at 4 ^o^C. The tissue was then washed 3× with PBS for 20 min and mounted on slides. A Zeiss 710 confocal microscope was used to image the tongue whole mounts. Z-stacks consisted of 50 stacks ~ at 1 μm step size, covering the tip of the taste bud to the gustatory nerve fiber core. Imaris software was used for the comprehensive analysis and 3-dimensional rendering of taste buds collected from whole-mount. The calculations of volume were done utilizing the surface function to discern the volume of taste buds based on Krt8 signal. The taste bud’s region of interest (ROI) was then used to quantify the innervation within the taste bud, as indicated by the tdTomato signal emanating from the gustatory fibers.

### Tongue imaging apparatus

A custom 3-D printed apparatus to stabilize the head and extrude the tongue was created with tinkerCAD software. The tongue holder is 2 cm by 4 cm and the hole for the tongue is a few millimeters in diameter. There are 2 additional holes for 2 metal screws to position and secure the mouse head. Magnets are attached to the screws and metal stage to stabilize the apparatus. The coverslip keeps the tongue flat to promote 3-D reconstruction of the gustatory fibers with the 2-photon microscope.

### Anesthesia and preparation of the mouse for imaging

Mice were given an initial IP injection of 10 mg/kg ketamine and 0.1 mg/kg xylazine (Covetrus). An IP injection was also given prior to positioning the mouse in the imaging apparatus to maintain fluid levels during imaging. The mouse was placed on his/her back and the tongue was inserted in the opening of the tongue holder using blunt forceps. After the tongue was placed through the opening, the coverslip was placed on the surface of the tongue. Initially, a 10× Olympus lens was used to spatially recognize that the same taste bud was being imaged. Once the correct bud was identified, a 40× water immersion lens was used to image the gustatory fibers. After each imaging session, the mouse was given an IP injection of Atipamezole (Covetrus) (1mg/kg) to reverse the effects of the sedative.

### Two-photon imaging

Two-photon excitation microscopy imaging was used to obtain 3-D images of a live mouse tongue, a Bruker system with Prairie View software. A laser strength of approximately 400 mV and a wavelength of 1100 nm was used to visualize the tdTomato-labeled nerve fibers innervating the tongue. The same PMT and imaging strength were used each day to ensure no variation due to the setting changes. Images were collected at 1024 × 1024 pixels, resonance galvo, with resonance averaging at 2 frames. This resonance setting was used because it takes a faster image which is critical due to our time constraints of the sedative. *Z* stacks were collected at 5 μm intervals covering approximately a volume of 100 µm, cubed. Typically, most of the taste bud is imaged within the first 50 µm of the *Z*-axis, but additional images were collected toward the base of the bud to help orient the 3-d volume as well as confirm that the same bud had been imaged day to day.

### Three-dimensional image analysis

ImageJ and Imaris were utilized for data analysis and 3-D reconstruction of 2-photon images. ImageJ software was used to obtain a volume measurement of the fibers in pixels, cubed. First, a consistent threshold setting was used in ImageJ to minimize background fluorescence/autofluorescence. Then, ImageJ was used to extract the region of interest and remove extraneous pieces that were not included in the analysis. This region of interest included the bottom portion of the bud that comprises the dense papilla core, excluding the gustatory fibers beyond the basal lamina. A volume calculator in ImageJ was utilized to calculate the volume of fibers innervating each taste bud from each section of the *z*-stack. Imaris software was used to create 3-D reconstructions of the innervating fibers after ImageJ was used to select the region of interest. The “surface volume” setting was applied to the z-stack. This setting finds the edges of 3-D images and renders an outline of the 3-D image.

## Results

### Gustatory fibers are reduced in fungiform and circumvallate taste buds within 4 days of CYP treatment

**Fig. 1:**
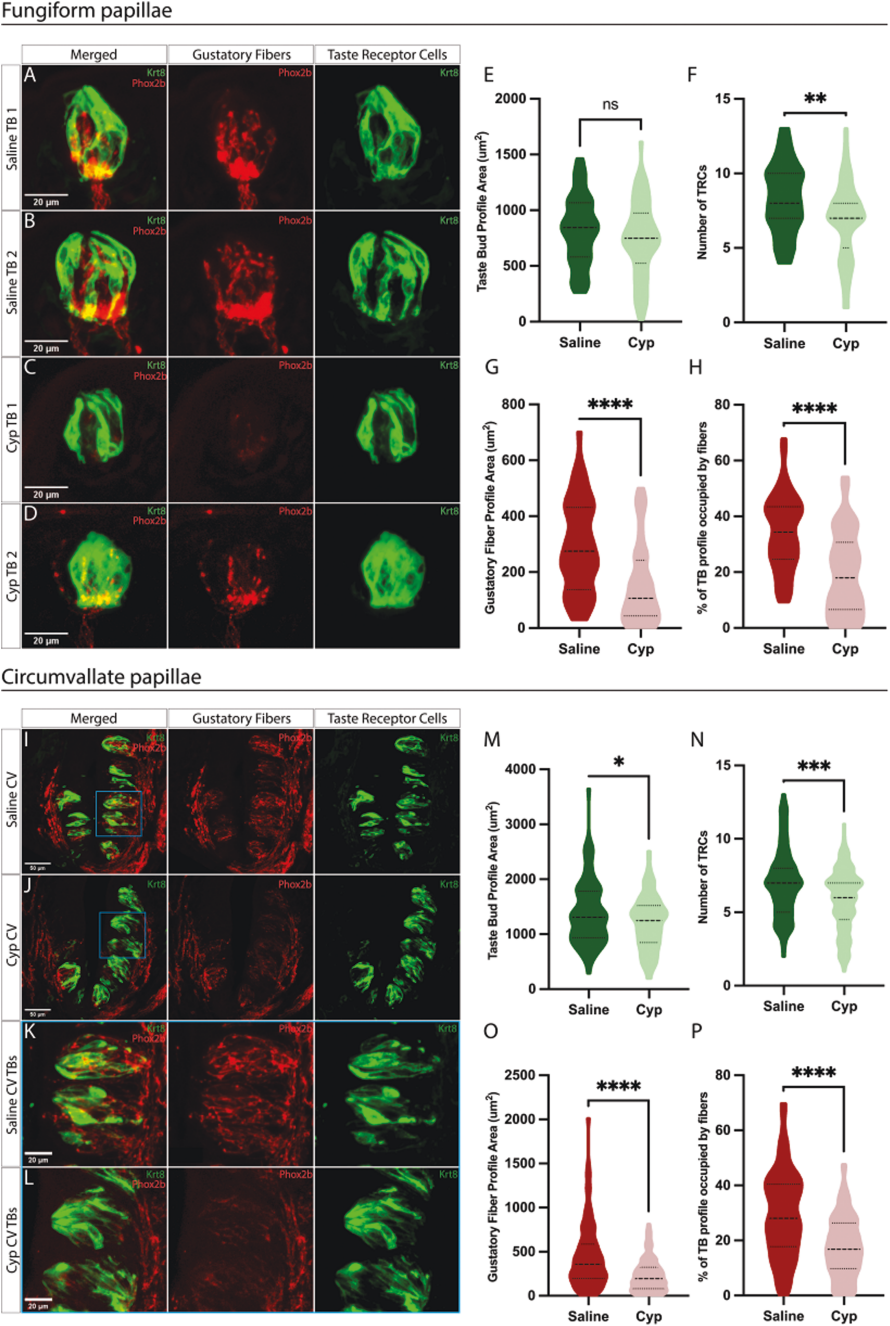
Gustatory fibers are reduced in fungiform and circumvallate taste buds within 4 days of CYP treatment. A, B. Saline: Fungiform tongue sections showing a single taste bud, stained for Krt8 (green) for TRCs along with Phox2b-Cre;tdTomato expression (red) for gustatory fibers from mice 4 days injected with saline. C, D. CYP: Fungiform tongue sections showing a single taste bud, stained for Krt8 (green) for taste receptor cells along with Phox2b-Cre;tdTomato expression (red) for gustatory fibers from mice 4 days injected with CYP (100mg/kg). E. Violin plot quantification of the area of Krt8 within taste bud profile area for saline vs CYP, no significant difference was seen (*N* = 60 TBs per group, 6 mice per group; ns = *P* > 0.05). F. Violin plot quantification of the average number of TRC cells labeled for Krt8 within the taste bud profile area for saline vs CYP, showing a decrease of TRCs within CYP-treated mice (*N* = 60 TBs per group; **P < 0.005). G. Violin plot quantification of the area of tdTomato-labeled fibers within the taste bud profile area for Saline vs CYP. Gustatory fiber area within the taste bud profile areas for CYP-treated mice was significantly reduced (*N* = 60 TBs per group; ****P < 0.00005). H. Violin plot quantification of the percentage of Phox2b-Cre;tdTomato area within the taste bud profile area for saline vs CYP. The percentage of gustatory fibers innervating the fungiform taste buds of CYP-treated mice was significantly decreased (*N* = 60 TBs per group; ****P < 0.00005). The Mann–Whitney *U* test was used to show the significance of each graph. I, J. Confocal images of circumvallate tongue sections focused on a single CV trench, immunolabeled using Krt8 (green) for taste receptor cells along with tdTomato expression (red) for gustatory fibers from mice 4 days after either saline injection (I) or CYP (J). K, L. Zoomed-in image of region located in the blue square for both saline and CYP CV. M. Violin plot quantification of the area of Krt8 within taste buds for saline vs CYP, showing a decrease in the gustatory fiber area within the taste buds profile area for CYP-treated mice (*N* = 111 to 113 TBs per group, 6 mice per group; *P < 0.05). N. Violin plot quantification of the average number of TRC cells labeled for Krt8 within the taste bud profile area for saline vs CYP, showing a minor decrease in the TRCs in CYP-treated mice (*N* = 111 to 113 TBs per group; ***P < 0.0005). O. Violin plot quantification of the area of gustatory fibers within taste buds for saline vs CYP, showing a decrease in the area of gustatory fibers within the taste bud profile area (*N* = 111–113 TBs per group; ****P < 0.00005). P. Violin plot quantification of the percentage of Phox2b-Cre;tdTomato area within the taste bud profile area for saline vs CYP, showing a decrease in the percentage of fibers innervating taste buds in CYP-treated mice (*N* = 111 to 113 TBs per group; ****P < 0.00005). The Mann–Whitney *U* test was used to show significance (*P < 0.05).

It is well-established that a single treatment of cyclophosphamide causes a transient loss of taste receptor cells within lingual taste buds (Delay et al. 2019). To determine whether the gustatory neurons are also affected by CYP treatment, we used an approach similar to Mukherjee and Delay (2011), Mukherjee et al. (2013, 2017), injecting a single dose of CYP. We used 12 Phox2b-Cre;tdTomato (Ohman-Gault et al. 2017) double transgenic mice to label gustatory fibers with tdTomato fluorescent protein. A single dose of CYP (100 mg/kg, IP) or saline was injected and then TRCs and neuronal fiber innervation were examined after 4 days. Fungiform and circumvallate taste bud tissue was collected and analyzed from CYP and saline treatment groups using anti-Keratin8 (Krt8) staining to label mature taste receptor cells in addition to the gustatory fibers labeled with tdTomato (Figure 1).

Consistent with previous reports (Mukherjee and Delay 2011; Mukherjee et al. 2013, 2017), we found that CYP treatment reduces the average number of TRCs within the fungiform taste buds (Fig. 1F; median CYP: 7 TRCs vs saline: 8 TRCs; *P* < 0.05) and circumvallate taste buds (Fig. 1N; median CYP: 6 TRCs vs saline: 7 TRCs; *P* < 0.05). There was a small, but significant decrease in the taste bud profile area in circumvallate taste buds (Fig. 1M), but this decrease was not significant in fungiform taste buds (Fig. 1E). Importantly, we found a significant reduction in the area of Phox2b-Cre;tdTomato labeled gustatory fibers within both fungiform (Fig. 1G; Median CYP: 106.8 μm^2^ vs saline: 275.3 μm^2^; *P* < 0.05) and circumvallate taste buds (Figure 1O; median CYP: 196.2 μm^2^ vs saline: 357.7 μm^2^; *P* < 0.05). Normalizing to the size of the taste bud, the percentage of fibers innervating the taste buds was significantly reduced in CYP-treated mice (Figure 1H; fungiform, median CYP: 17.94% vs saline: 34.32%; *P* < 0.05 Fig. 1P, Circumvallate; median CYP: 16.84% vs saline: 28.05%; *P* < 0.05). Since the reduction of gustatory fiber innervation is observed in both the fungiform and circumvallate papillae, this indicates that CYP affects both the chorda tympani nerve which innervates the anterior tongue as well as the glossopharyngeal nerve that innervates the posterior tongue.

### Fungiform taste bud density and gustatory fiber volume decrease within 4 days of CYP treatment

**Fig. 2.**
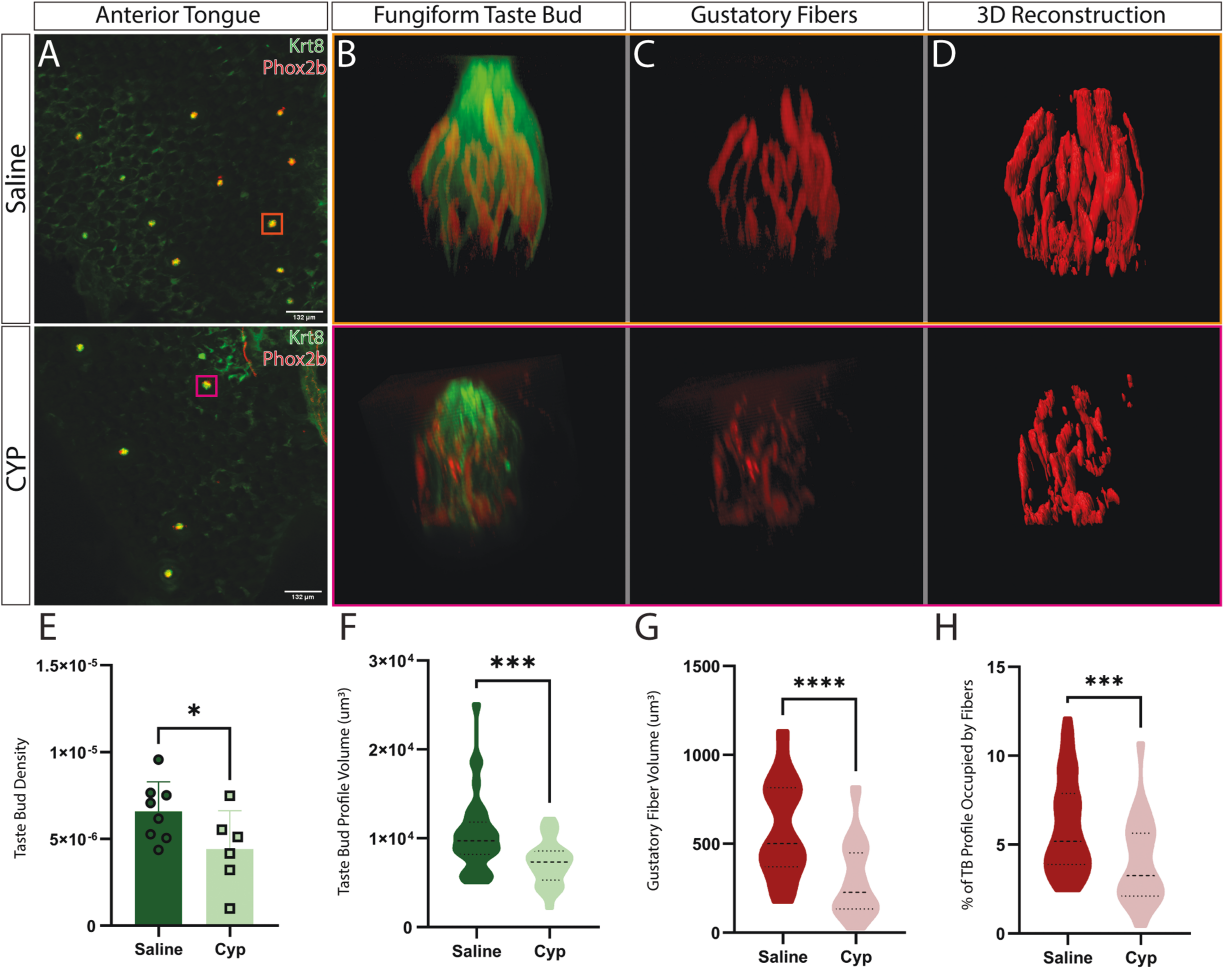
Fungiform taste bud density and gustatory fiber volume decrease within 4 days of CYP treatment. Top: Saline, Bottom: CYP A. Surface image of the anterior portion of tongue, stained with Krt-8 (Green) to show taste receptor cells, and showing Phox2b-Cre; tdTomato expression (Red) for gustatory fibers. B, C. A representative taste bud from each group showing gustatory fibers within the bud, along with an isolated reconstruction of only gustatory fibers. D. 3D-reconstruction of gustatory fibers within taste buds for both treatment groups. E. Quantification of the density of taste buds within the first 2 mm of the anterior tip of the tongue for saline vs CYP, showing a significant decrease in the anterior taste buds for CYP-treated mice (*N* = 6 to 8 mice per group; *P < 0.05). F. Violin plot quantification of the volume of the taste buds for saline vs CYP, showing a decrease in the bud volume for CYP (*N* = 30 TBs per group, 6 mice per group; ***P < 0.0005). G. Violin plot quantification of the volume of gustatory fibers within taste buds for saline vs CYP, showing a decrease of gustatory fibers within the taste buds (*N* = 30 TBs per group; ****P < 0.00005). H. Violin plot quantification of the percentage of gustatory fiber volume within the volume of the taste bud for saline vs CYP, showing a decrease in the percentage of fibers within the taste buds of CYP-treated mice (*N* = 30 TBs per group; ***P < 0.0005). The Mann–Whitney *U* test was used to show significance.

Our initial experiments show a remarkable decrease in the gustatory fibers innervating fungiform taste buds within 4 days of CYP treatment. To understand the full scope of the CYP effects on gustatory neurons innervating fungiform taste buds, we performed a more in-depth analysis using whole-mount staining of the anterior tongue. 12 Phox2b-Cre; tdTomato mice were injected with either CYP or saline, and the anterior tongue was collected 4 days post-treatment. Anti-Krt8 was used to visualize/analyze the taste receptor cells. Examining the 2 mm tip of the anterior tongue, we found a decrease in the taste bud density 4 days post-CYP treatment (Fig. 2A and E; median CYP: 4.635e-6 vs saline: 6.612e−6; *P* < 0.05). We also found a decrease in the total volume of the fungiform taste buds in CYP-treated mice (Fig. 2B and F; Median CYP: 9705 μm^3^ vs saline: 7325 μm^3^; *P* < 0.05). Similarly, the gustatory fibers also decreased in volume after CYP treatment (Fig. 2C, D, and G; median CYP: 226.5 μm^3^ vs saline: 501.3 μm^3^; *P* < 0.05). Normalizing to taste bud volume, the percentage of fibers innervating CYP-treated taste buds was reduced compared to saline controls (Fig. 2H; median CYP 3.255% vs saline: 5.185%; *P* < 0.05). This demonstrates that CYP not only causes TRC death but also reduces gustatory fiber innervation in taste buds to a similar or even greater degree.

### Intravital imaging of individual fungiform taste buds reveals the morphology and dynamics of gustatory fiber innervation day-to-day

**Fig. 3.**
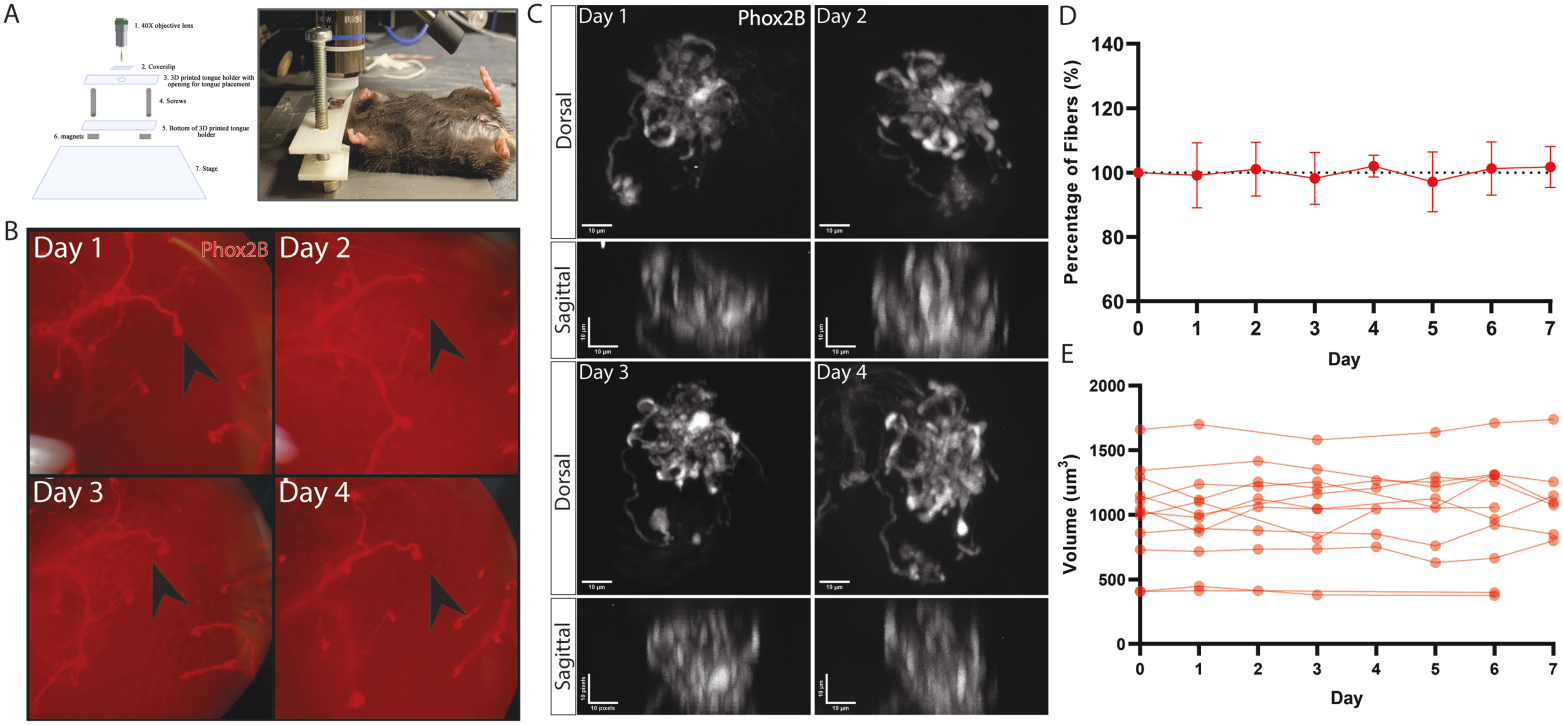
Using intravital imaging of individual fungiform taste buds to visualize the morphology and measure the volume of gustatory fibers day to day. A. Schematic of 2-photon Imaging of the anterior fungiform taste buds from Phox2b-Cre;tdTomato mice using a 3D-printed head holder. B. Tongue map within a single mouse over the course of 4 days of imaging the anterior tongue. Fibers are illuminated in red with an individual taste bud indicated by the black arrow. C. A single taste bud was tracked for 4 days, showing maximum projection of the z-stack for dorsal and re-sliced maximum projection of sagittal images. D. Quantification of the percent change of gustatory fiber volume within taste buds over the course of 8 days, normalized to day 0, showing that gustatory fiber volume remains consistent over multiple days of imaging (*N* = 12 TBs). E. Quantification of the gustatory fiber volume of individual taste buds over the course of 8 days (*N* = 12 TBs).

Immunohistochemistry, while useful in identifying large changes in the makeup of the taste bud, is unable to visualize how individual taste buds are affected by treatment over time. To see the changes over time in individual taste buds, we used 2-photon intravital imaging of the anterior tongue of anesthetized Phox2b-Cre;tdTomato mice. We used a custom 3D-printed head harness/tongue holder (Fig. 3A) to identify and track the same taste buds over the course of multiple days (Fig. 3B and C). Z-stack images were taken from the apical tips of the gustatory fibers at the papillae pore opening through the basal plexus/core where the fibers coalesce, and into the subepithelium (approximately 60 µm in depth). Only the area within the taste bud proper, inclusive of the core was used in the volume analysis. Similar to Whiddon et al.’s report (Whiddon et al. 2023), we find that although individual gustatory fibers are dynamic over time, the volume of the innervating gustatory fibers remains consistent over the course of multiple days, fluctuating only between 95% and 105% of their original volume (Fig. 3D). Individual taste buds vary in the volume of fiber innervation, but maintain that size over 8 days of daily imaging (Fig. 3E). These results indicate that the average volume of innervation in untreated taste buds remains relatively unchanged over time, and that repeated 2-photon imaging does not affect their morphology, volume, or the quality of tdTomato labeling.

### CYP treatment changes the morphology and volume of gustatory fiber innervation

**Fig. 4.**
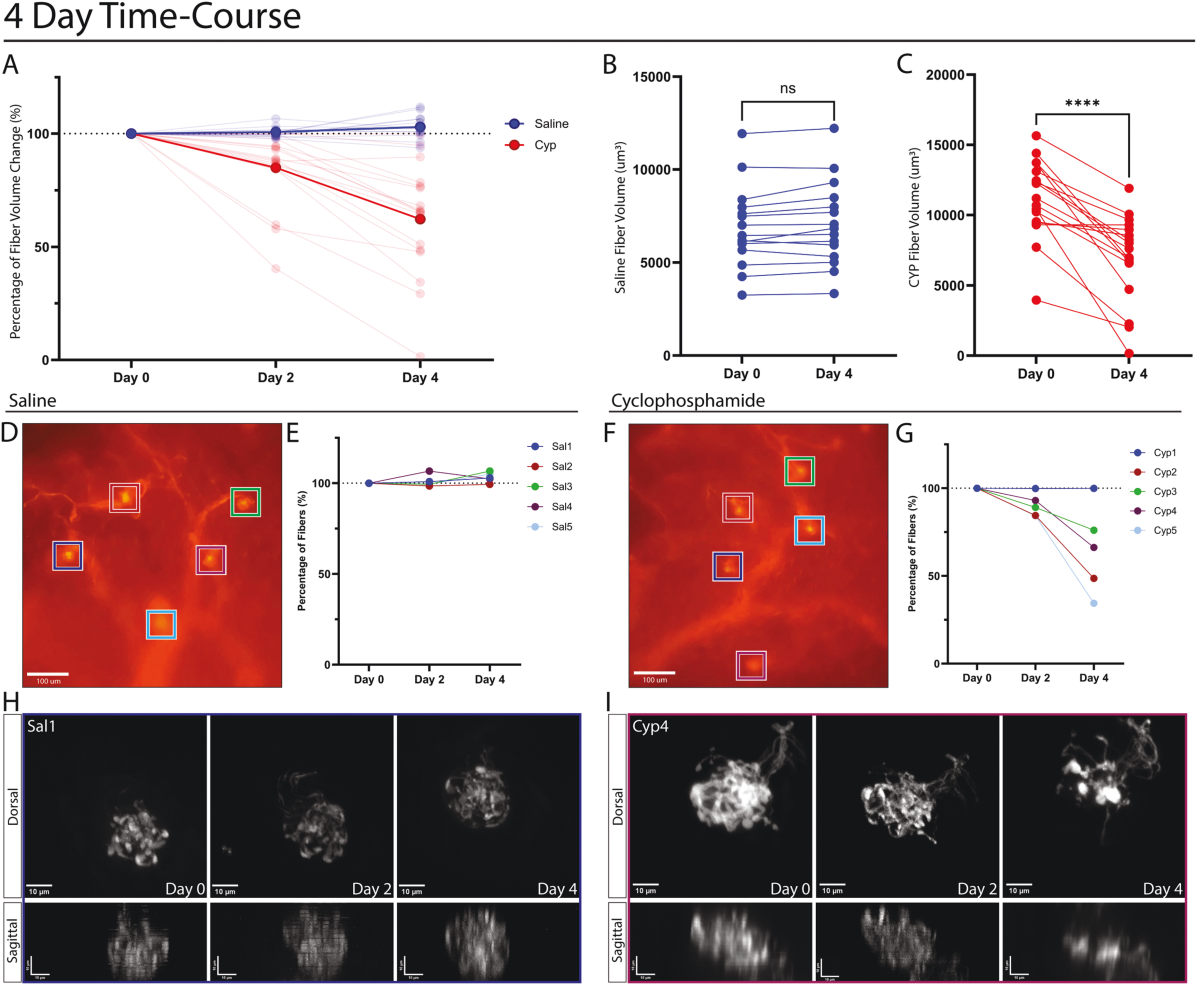
Intravital imaging reveals decreased volume of gustatory fiber innervation after CYP treatment. A. Quantification of the percentage change per taste bud for saline vs CYP mice over a 4 -day period. Solid line displays the averages within each treatment group. Transparent lines show individual taste bud values. Taste buds from CYP-treated mice show a decrease in their gustatory fiber volume over the course of treatment. B, C. Quantification of the volume of the taste buds for mice injected with CYP and saline, each line represents one taste bud, showing a decrease in taste buds’ gustatory fiber volume 4 days after CYP treatment. Saline-treated taste buds remain consistent in their volume (*N* =15 to 17 TBs per group). Wilcoxon ranked sum tests were used to show significance (*P < 0.05). D. Tongue map from a saline-injected mouse, showing 5 tracked taste buds in different colors. E. Quantification of the percent change of gustatory fiber volume per taste bud for a saline-treated mouse over 4 days. F. Tongue map from a CYP injected mouse, showing 5 tracked taste buds in distinct colors. G. Quantification of the percent change of gustatory fiber volume per taste bud for a CYP-treated mouse over 4 days. H. Sal1 (Blue) taste bud from a saline-treated mouse tracked for 4 days, showing the maximum intensity projection of the z-stack for dorsal, and re-sliced maximum projection for sagittal images. I. Cyp4 (purple) taste bud from a CYP-treated mouse tracked for 4 days, showing the maximum intensity projection of the z-stack for dorsal, and re-sliced maximum projection for sagittal images.

To determine how gustatory nerve fibers change over the course of CYP treatment, 6 Phox2b-Cre;tdTomato mice were imaged to identify distinct taste buds. These mice were then separated into 2 groups, the treatment group was injected with CYP and the control group was injected with saline. Z-stacks of tracked taste buds were imaged 2- and 4-days post-treatment (Fig. 4). The gustatory fibers of saline-injected mice (16 buds from 3 mice) are consistent in their volume over the course of imaging (Fig. 4B). The CYP-treated group (17 buds from 3 mice) showed a significant decrease in the volume of gustatory fibers within individual taste buds 4-days post-treatment (Fig. 4C). The gustatory fibers began to decrease 2 days after CYP injection. Figure 4A displays the percent change of Phox2b-Cre;tdTomato fiber volume in individual taste buds over time for both CYP-treated and control mice.

To examine the CYP-induced fiber loss more closely, Fig. 4D–I show one representative saline- and one CYP-treated mouse. Taste maps were taken to track individual taste buds over the course of treatment (Fig. 4D and F). As seen in our previous results, fiber volume remained consistent after multiple days of imaging in saline-injected mice (Fig. 4E). The taste buds of CYP-treated mice were variable in response to the treatment, with some of the taste buds showing little change in fiber volume or morphology after treatment, and others showing a significant decrease in their volume and changes in morphology (Fig. 4G). For example, one taste bud showed only a minor decrease in innervation 2 days post-treatment, but by day 4, there was a major loss in the gustatory fibers within the taste bud, showing a loss in *z*-height (Fig. 4I, Supplementary Video 1). There was no obvious correlation between taste bud location or size and sensitivity to CYP treatment.

**Fig. 5.**
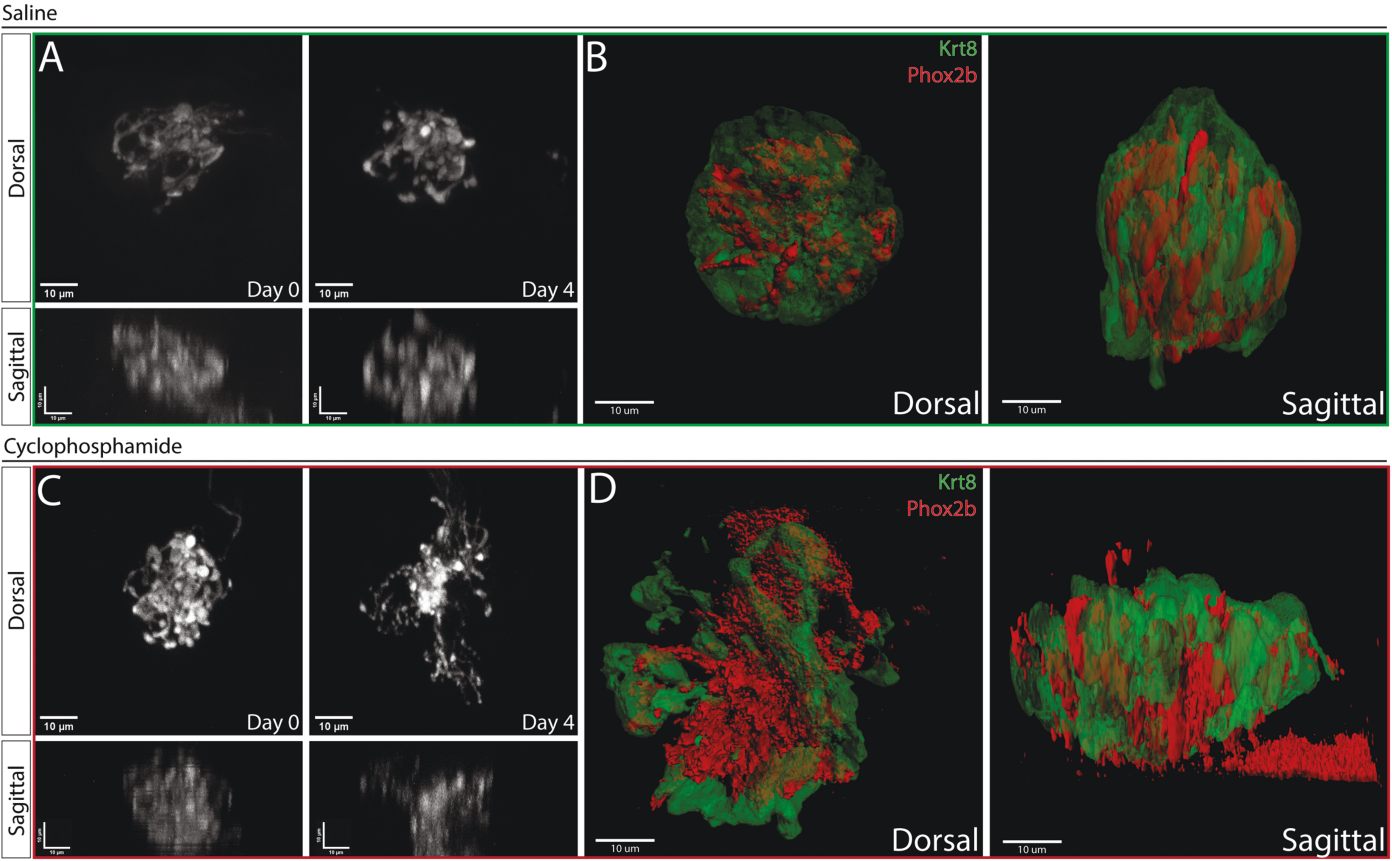
CYP changes the morphology of taste receptor cells and gustatory fibers. A. A taste bud from a saline-treated Phox2b-tdTomato mouse (Sal3, green outline) imaged at 0 and 4 days after saline injection, showing the maximum intensity projection of the z-stack for dorsal, re-sliced maximum intensity projection of sagittal plane. B. 3D-reconstruction of the same taste bud after whole mount immunolabeling with Krt8. The tissue was collected 4 days after saline injection, taste receptor cells labeled with Krt8 are shown in green, and gustatory fibers shown in red, dorsal and sagittal planes of the taste bud are displayed. C. A taste bud from a CYP-treated Phox2b-Cre;tdTomato mouse (Cyp2, red outline) tracked over 4 days, showing the maximum projection of the z-stack for dorsal, re-sliced maximum projection of sagittal images for 0 and 4 days. D. 3D-reconstruction of the same taste bud after whole mount immunolabeling with Krt8. The tissue was collected 4 days after CYP injection, taste receptor cells labeled with Krt8 are shown in green, and gustatory fibers shown in red, dorsal and sagittal planes of the taste bud are displayed.

For a subset of mice, tongue tissues were isolated 4 days after treatment and then stained with anti-Krt8 using the whole-mount IHC protocol as previously described. Taste buds from saline-treated mice showed consistent gustatory fiber volume from the 2-photon imaging (Fig. 5A). These taste buds remained intact, with the gustatory fibers innervating the TRCs (Fig. 5B). Figure 5C shows a representative taste bud from a CYP-treated mouse which showed a decrease in volume by 4 days post-treatment, with the fibers spreading out from the fiber core (Fig. 5C). Examining whole-mount staining of taste buds reveals their diameter to have grown in some cases, with the gustatory fibers appearing to be extending from the core to follow the remaining TRCs (Fig. 5D). All the results together show a significant decrease in gustatory fiber volume after CYP treatment, although it is variable to how severe this loss is dependent on the taste bud.

### Dynamics of the loss and recovery of gustatory fiber innervation within cyp-treated taste buds

**Figure 6.**
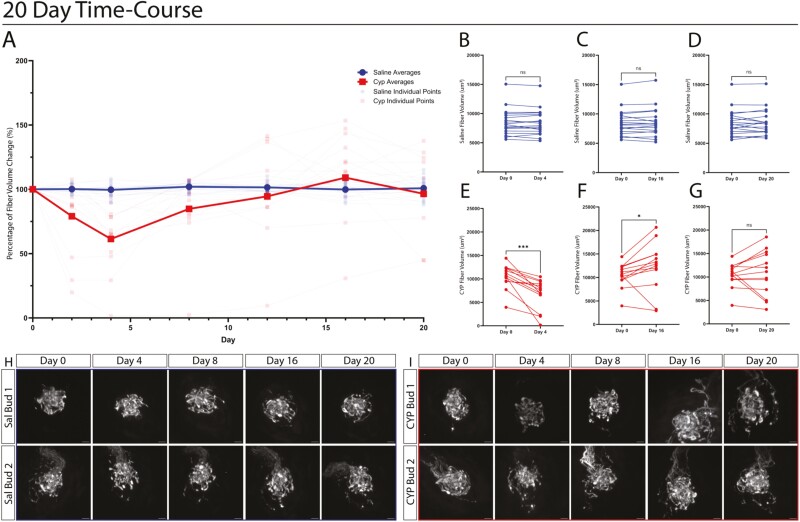
Intravital imaging over a 20-day time course reveals an initial decrease in gustatory fiber volume followed by their recovery. A. Quantification of the percentage change in gustatory fiber volume per taste bud for saline vs CYP-treated mice over a 20-day period. Solid lines showing the averages, and transparent lines showing individual taste buds. Taste buds of CYP-treated mice show a decrease in the percentage of gustatory fiber volume as previously described by day 4. An increase in volume is observed on day 16, and volumes recover by day 20. B–D. Quantification of the gustatory fiber volume within the taste buds for mice injected with saline. Each line represents one taste bud. The gustatory fiber volume of saline-treated mice remains consistent over the course of treatment. Days 4, 16, and 20 are compared to day 0 (*N* = 18 TBs per group). The Wilcoxon ranked sum test was used to test for significance (*P < 0.05). E–G. Quantification of the gustatory fiber volume within the taste buds of CYP-treated mice. Each line represents one taste bud, showing a decrease in taste buds’ gustatory fiber volume 4 days after CYP treatment. By day 16, the gustatory fiber volume is increased when compared to day 0, beginning to recover to pre-injection levels by day 20. The Wilcoxon ranked sum test was used to test for significance (*P < 0.05). H. Examples of 2 taste buds from saline-treated mice, showing images over the 20-day course of treatment (scale bar = 10 μm). I. Examples of 2 taste buds from CYP-treated mice, showing images over the 20-day course of treatment (scale bar = 10 μm).

In previous studies investigating the effects of CYP on the taste system, taste receptor cell populations (Mukherjee et al. 2017) and taste detection thresholds (Mukherjee and Delay 2011; Mukherjee et al. 2013) largely recover to baseline levels by 16 to 20 days after treatment. To determine if gustatory fibers recover after CYP treatment in a similar time frame, we imaged both saline and CYP injected mice over the course of 20 days. The gustatory fibers of saline-injected mice (16 buds from 3 mice) remained consistent in their volume over the course of imaging (Fig. 6A–D). Similar to our previous results, the CYP-treated mice (13 buds from 2 mice) showed a significant decrease in their gustatory fiber volume 4-days post-treatment, which appears to be the peak of volume loss (Fig. 6A and F). The CYP-treated gustatory fibers begin to recover after 4 days, transiently over-innervating the buds by day 16 (Fig. 6E). By 20 days, a new pattern of innervation is established, that is on average similar to the initial innervation pattern (Fig. 6G). Figure 6H and I shows 2 representative taste buds from saline- and CYP-treated mice. As previously noted, the morphology of the taste buds of saline-treated mice remains largely consistent over multiple days of imaging (Fig. 6H, Supplementary Video 1). In contrast, the taste buds of CYP-treated mice show a rapid change in morphology with a decrease in volume by day 4 (Fig. 6I, Supplementary Video 2). As the taste buds begin to recover, the addition of fibers surrounding the bud begins to appear by day 16 (Fig. 6I, Supplementary Video 2). By day 20, while the average volume of taste innervation returns to baseline levels in CYP-treated mice, many individual taste buds have adopted new innervation patterns (Fig. 6I, Supplementary Video 2).

## Discussion

Consistent with previous reports, we found that a single dose of 100 mg/kg cyclophosphamide produces a loss of taste receptor cells in the fungiform and circumvallate papillae within 4 days of treatment (Mukherjee and Delay 2011; Mukherjee et al. 2013, 2017). In addition, we find that gustatory fiber innervation in both taste papillae is also reduced within 4 days of CYP treatment. Intravital imaging of Phox2b-Cre;tdTomato labeled gustatory fibers innervating the fungiform taste buds revealed that some buds were more affected than others. Fibers changed their morphology, primarily showing a retraction of fibers from the apical parts of the bud, and splaying out or spreading of fibers at the core/base of the epithelium.

These results suggest that gustatory fibers are indeed sensitive to chemotherapy treatment, at least indirectly. Gustatory fiber volume is reduced within the taste bud proper by 4 days of treatment. This coincides with the loss of taste receptor cells within the bud. While the average reduction in fiber innervation is significant when averaged across animals, individual buds vary in the degree of severity. This variability was more obvious when observed in whole-mount preparations of the fungiform taste papillae and using 2-photon intravital imaging.

To the best of our knowledge, this is the first report detailing the effects of CYP on gustatory nerve fibers within taste buds. The closest comparable studies have used another agent, sonidegib, which is a hedgehog pathway inhibitor and is used to treat some basal cell carcinomas. Long-term treatment (25 to 28 days) of sonidegib (oral gavage 20 mg/kg) inhibits the maturation of taste receptor cells, and eventually eliminates the taste bud entirely (Donnelly et al. 2022). Although the chorda tympani nerve in sonidegib treated rodents is no longer sensitive to taste stimuli, mechanosensory, and thermosensory responses are maintained (Kumari et al. 2015, 2017, 2018). After 25 to 28 days of sonidegib treatment, the morphology of Phox2b-Cre;tdTomato labeled chorda tympani fibers change dramatically, reorganizing and expanding just under the fungiform papilla epithelium where the taste buds previously were (Donnelly et al. 2022). Our study uses a single dose of CYP, which has a less dramatic effect on the TRCs within the bud compared to long-term sonidegib. Nevertheless, we observed several examples of taste buds where the fibers also flatten and spread out at the basal side of the bud. In our experiments, this reorganization happens within 4 days of CYP treatment. In the sonidegib study, increased expression of GAP43 indicated that these fibers were in the process of remodeling. It will be interesting to test the time course of GAP43 expression in the fibers after CYP treatment during this period as well, or later in the recovery phase 8 to 20 days post-CYP.

Another recent study used 2-photon intravital imaging to track the dynamics of individual gustatory fibers in the taste buds (Whiddon et al. 2023). Similar to Whiddon et al., we also find that the terminal fibers within the taste bud are surprisingly dynamic. While our study does not attempt to track the dynamics of individual fibers innervating the bud as Whiddon does, we do see a remarkable amount of motility and change of the fibers within the bud, while the overall volume of innervation is maintained (at least for the saline-treated control mice). This study also used sonidegib (LDE225) treatment to perturb the taste buds. Interestingly, 10 days after LDE225 treatment, no major differences in the rate of terminal branch changes were reported compared to controls. And even 20 to 40 days after treatment, the dynamics of terminal arbors remained regardless of whether the taste bud had recovered or not. These data are somewhat inconsistent with Donnelly et al., but this may be due to a shorter sonidegib treatment window (10 days vs 25 days) or the fact that only individual nerve fibers were labeled, rather than the entire Phox2b + population (Donnelly et al. 2022). Together, these studies, and ours, highlight the usefulness and promise of intravital imaging to investigate important questions about the dynamics of taste bud remodeling in normal animals and in those where the taste system is perturbed.

Loss of taste function is also commonly reported by patients undergoing radiotherapy treatment for head and neck cancers. Fractionated and single-dose irradiation in mice reduces taste progenitor cell numbers and their proliferation, interrupting the supply of new taste receptor cells to taste buds(Nguyen et al. 2012; Gaillard et al. 2019). These studies did not investigate the effects of radiotherapy on the gustatory fibers within taste buds, but it will be important to see if both radio- and chemotherapies have similar or different effects. This will have important implications for future studies investigating the mechanisms by which gustatory fibers are lost/retracted from taste buds during cancer treatment.

Gaillard et al. suggest a role for Wnt/B-catenin and potentially other signaling pathways to maintain taste stem cell proliferation during radiation exposure and/or promote taste cell differentiation following radiation injury (Gaillard et al. 2019). Importantly, R-spondin is expressed and secreted by gustatory neurons and potentiates the Wnt pathway (Lin et al. 2021; Lu et al. 2022). R-spondin has also been shown to be the neuronal factor necessary for taste bud homeostasis (Lin et al. 2021; Lu et al. 2022). Interestingly, treatment with Rspo1 is protective for chemotherapy- and radiotherapy-induced oral mucositis (Zhao et al. 2009). This points to a potentially important role of gustatory neurons to produce R-spondin and promote the differentiation of taste stem cell populations to recover taste buds after insults like chemo and radiotherapy. It will be important to determine if the remodeling/migration of gustatory fibers toward the base of the taste bud after the CYP treatment that we observed serves a restorative function, stimulating the remaining stem cell populations to generate more mature TRCs and lingual epithelial cells.

Another important factor that may influence the loss and recovery of taste function after chemotherapy treatment is inflammation. Cyclophosphamide treatment in mice quickly induces TNF-α expression, especially by Type II TRCs. TNF-α levels peak at 8 to 24 h post-CYP injection, but persist at elevated levels for ~72 h (Sarkar et al. 2021). Pre-treatment with amifostine, a sulfhydryl drug that protects tissues during chemo- or radiation therapy, reduced the amount of CYP-induced TNF-α expression in taste buds (Sarkar et al. 2021), and has been shown to improve taste function in CYP-treated mice (Mukherjee et al. 2013). It is very likely that inflammation may also reduce the ability of gustatory neurons to properly innervate the TRCs within the taste buds or otherwise disrupt taste signaling, just as inflammation affects other peripheral sensory neurons such as nociceptors (Ruda et al. 2000; Xu and Huang 2002; Sommer and Kress 2004; Breese et al. 2005; Takeda et al. 2007). It will be important to understand the relationship between inflammation and gustatory neuron innervation both in the context of chemotherapy/radiotherapy treatment, and also for other inflammatory conditions that can affect the taste system including obesity, diabetes, and metabolic syndrome, among others.

In summary, we find that gustatory nerve fibers innervating taste buds are sensitive to cyclophosphamide treatment, reducing innervation of the taste buds within 2 to 8 days, but re-innervating the buds at normal levels by 20 days post-treatment. Individual taste buds in the fungiform papilla differ in their response to CYP, with some maintaining near-normal levels of innervation, and others losing the majority of their innervation within the taste bud proper. Future experiments will be needed to determine the mechanisms and sequence of events leading to fiber retraction as it relates to taste receptor cell loss within the buds. Fibers may begin to retract earlier than TRCs die, but perhaps subsequent to inflammatory signaling events. To further investigate if and how the taste buds fully regain function, it will be necessary to investigate the period between 8- and 20-days post-treatment more carefully, especially given that many chemotherapy patients report prolonged taste dysfunction long after their treatment concludes. It will also be important to determine if this effect is generalizable across multiple types of chemotherapy agents and radiation-based treatments for cancers.

## Supplementary Material

bjae010_suppl_Supplementary_Figure

bjae010_suppl_Supplementary_Videos_1

bjae010_suppl_Supplementary_Videos_2

## Data Availability

The data described in this article will be shared on request to the corresponding author (LJM).
