# Supplementary figures and images for: Cyclophosphamide induces the loss of taste bud innervation in mice
Source: Chem Senses. 2024 Feb 29;49:bjae010. doi: 10.1093/chemse/bjae010 (PMC10929424; doi:10.1093/chemse/bjae010)

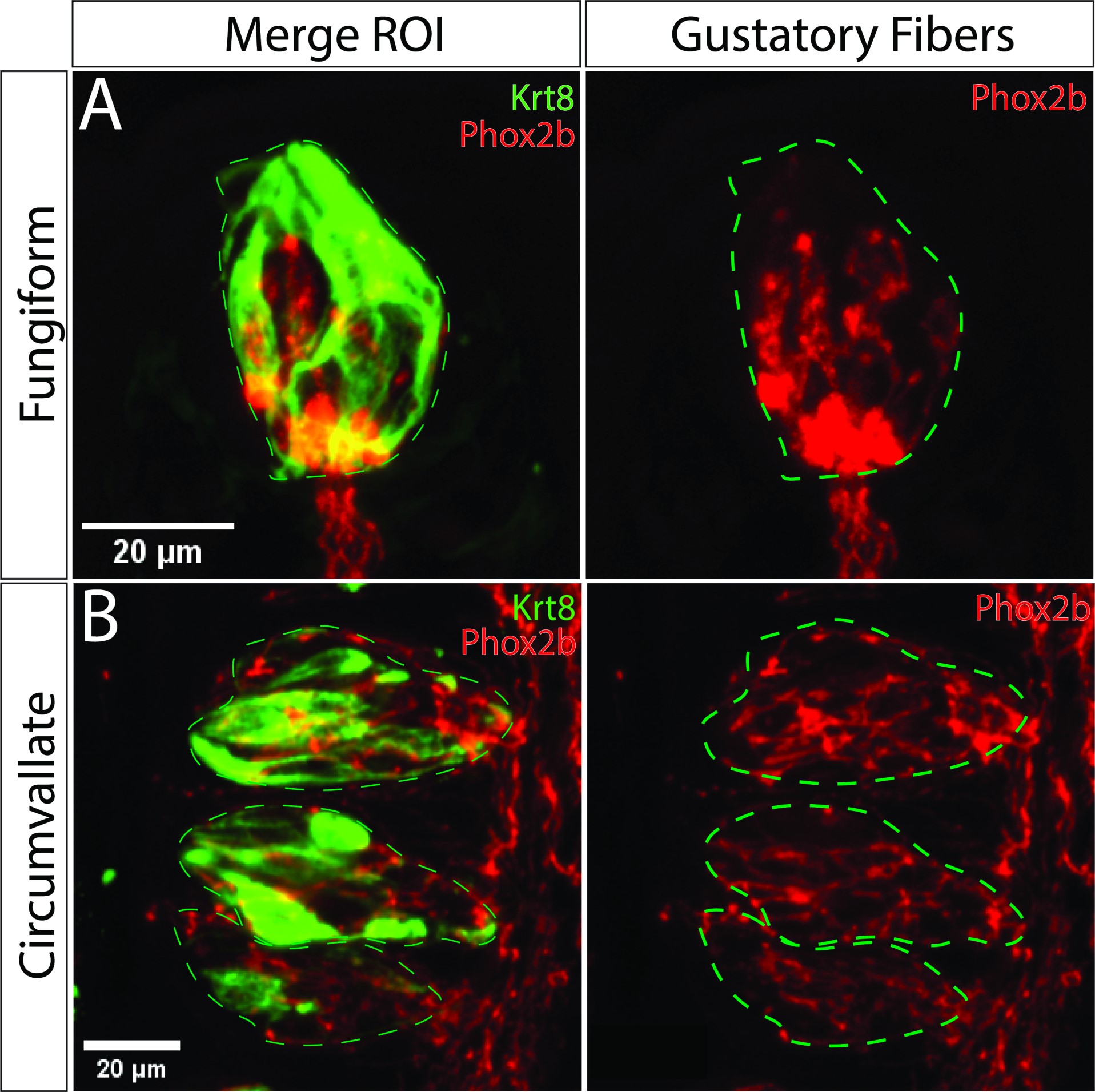

Supplement: bjae010_suppl_Supplementary_Figure [file bjae010_suppl_supplementary_figure.jpeg]
